# Persistence with medical treatment for Wilson disease in China based on a single center’s survey research

**DOI:** 10.1002/brb3.2168

**Published:** 2021-05-05

**Authors:** Zhi‐Hua Zhou, Yun‐Fan Wu, Yan Yan, Ai‐Qun Liu, Qing‐Yun Yu, Zhong‐Xing Peng, Gong‐Qiang Wang, Ming‐Fan Hong

**Affiliations:** ^1^ Department of Neurology The first affiliated hospital School of Clinical Medicine of Guangdong Pharmaceutical University Guangzhou China; ^2^ The Second School of Clinical Medicine Southern Medical University Guangdong Second Provincial General Hospital Guangzhou China; ^3^ Wilson Disease Centre Hospital Affiliated to Institute of Neurology Anhui University of Chinese Traditional Medicine Hefei China

**Keywords:** medicine adherence, nonpersistence with drug treatment, persistence with drug treatment, Wilson's disease

## Abstract

**Background:**

Wilson's disease (WD) is one of the few hereditary diseases that can be successfully treated with medicines. We conduct this survey research to assess treatment persistence among patients with WD and try to identify what factors affect the treatment persistence.

**Methods:**

We employed WeChat which is the most popular social software in China to carry out this anonymous questionnaire research. The questionnaire included medication adherence scale. We also collected available medical records related to demographic and clinical characteristics. All the patients were divided into group of persistence with drug treatment (PDT) and nonpersistence with drug treatment (n‐PDT).

**Results:**

We collected 242 qualified questionnaires. Only 66.5% of patients were PDT during the mean 12.6 years of follow‐up. In PDT group, better outcomes were observed: improvement (78.3%) and no change (16.1%) versus those in n‐PDT (55.6%; and 28.4%, respectively). In PDT group, only nine patients deteriorated (6.8%) in comparison with 13 patients in n‐PDT (16.0%). The adverse events (AEs) in PDT group were significantly less than those in n‐PDT group. There were no significant differences in clinical type, gender, age, education level, and family knowledge about WD between the two groups. There were significant differences in AEs and family position toward treatment.

**Conclusion:**

Medication Adherence of Chinese WD patients was low. One third of the patients (33.5%) were unable to PDT, and it had an important negative effect on clinical outcome. AEs and family support had an important impact on treatment persistence.

## Graphical abstract

Medication Adherence of Chinese WD patients was low. One third of the patients (33.5%) were unable to persistence with drug treatment, and it had an important negative effect on clinical outcome. Adverse events and family support had an important impact on treatment persistence.

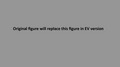

## 1 BACKGROUND

Wilson's disease (WD) is a rare, autosomal recessive disease of abnormal copper metabolism which was described firstly by the British neurologist Kinnier Wilson in 1912 (Compston, 2009). The causative gene of WD is ATP7B, which encodes a copper‐transporting ATPase in the liver and functions as a copper‐dependent P‐type ATPase (European Association for Study of Liver, 2012; Berghe et al., 2009). Major clinical manifestations of WD include neuropsychology, damaged liver, and renal symptoms, as well as Kayser–Fleischer rings (K–F rings) of the cornea (European Association for Study of Liver, 2012). So WD needs a lifelong medication under the current medical conditions.

Recently, the drug treatment of WD has made some progress (Litwin et al., 2019). So far available therapies for WD are metal chelating agents [D‐penicillamine (PCA) and trientine] and zinc salts which can competitively inhibit copper absorption in the intestine (European Association for Study of Liver, 2012). At present, the most commonly used metal chelating agents are PCA which was the first successful treatment of WD by Walshe in 1956 (Walshe, 2003) and trientine in 1969 (Walshe, 1982). But, recent studies have shown that some patients can be worsened following metal chelating agents, especially PCA (Ranjan et al., 2015; Kalita et al., 2014, 2015). Recent study demonstrated one quarter of WD patients could not take anti‐copper treatment regularly, and this had an important negative effect on clinical outcome (Maselbas et al., 2019a). Persistence with treatment resulted had significantly better clinical outcome than non‐persistence treatment in WD (Maselbas et al., 2010).

Recently, the study of WD has made great progress in China (Xie & Wu, 2017). Besides PCA and zinc gluconate, there also have dimercaptosuccinic acid (DMSA) and Gandou tablet (GDT; Chinese patent medicine) which were recommended by Guideline for diagnosis and treatment of Wilson disease in China (Parkinson's Disease & Dyskinesia Group of Neurology Branch of Chinese Medical Association & Neurogenetics Group of Neurology Branch of Chinese Medical Association, 2008). In China, the choice of medicines for WD patients is more abundant than that in western countries. So we conduct this survey research to assess treatment persistence among patients with WD and try to identify what factors affect the treatment persistence with WD in our center.

## 2 METHODS

### 2.1 Patients and assessments

We employed WeChat which is the most popular social software in China to carry out this anonymous online questionnaire research. All the respondents guaranteed to answer the questions truthfully. We collected 245 questionnaires over 10 days. All the patients were treated from July 2005 to December 2019 in department of neurology, the first affiliated hospital of Guangdong Pharmaceutical University. All patients met the diagnostic criteria for WD (Ferenci et al., 2003). Patients were divided into two groups, group of hepatic and group of neurological. Group of hepatic was defined as hepatic presentation at the time of diagnosis of exclusively hepatic symptoms and signs and the absence of neurologic symptoms and signs as confirmed by careful neurologic examination. Group of neurological was defined as neurologic presentation and the absence of hepatic symptoms at the time of diagnosis. Just after diagnosis, the patient received anti‐copper treatment with PCA or DMSA (PCA and DMSA were replaced each other every 3 months), zinc gluconate, GDT (Chinese patent medicine), and the four main treatments used in our hospital.

### 2.2 Questionnaire

The questionnaire included questions based on already validated scales: 8‐item Morisky Medication Adherence Scale (MMAS‐8; Morisky et al., 2008), 10‐point Medication Adherence Visual Analog Scale (MA‐VAS; Nau et al., 2007), a self‐report measure of adherence. All the patients were also divided into two groups, group of persistence with drug treatment (PDT) and group of nonpersistence with drug treatment (n‐PDT; Maselbas, Czlonkowska, et al., 2019), after detailed interview, and analysis of patient questionnaires, refills, and drug consumption. Patients were classified into nonpersistent if they had at least one reported break of more than 3 months or a minimum of two breaks lasting longer than 2 months within the period of treatment (Maselbas, et al., 2019).

In addition to drug adherence investigations, we also collected available medical records related to demographic and clinical characteristics. We are particularly focused on prescribed medication, clinical outcome, and reported adverse events (AEs). The principles of the Declaration of Helsinki were followed. Written informed consents were obtained from all participants.

### 2.3 Statistical analysis

Statistical analysis was performed using SPSS version 16.0 for Windows (SPSS IBM). Collected data were reported with descriptive statistics and analyzed by two‐tailed Fisher's exact test, chi‐square test, Mann–Whitney test, and unpaired Student's *t* test were appropriate. A *p* < .05 was considered statistically significant.

## 3 RESULTS

### 3.1 Demographic and clinical characteristics

Of the 245 patients completed the online questionnaire, due to incomplete data (three patients) in this survey, the final analysis was conducted on 242 subjects. The demographic and clinical characteristics of 242 patients are shown in Table 1. Of the 242 patients, 113 were female, accounting for 46.7% and 129 were male, accounting for 53.3%. The ratio of female and male was 0.88:1. The mean time from symptom onset was 16.2 ± 8.7 years and the mean age at diagnosis was 19.1 ± 7.1 years. So we roughly estimate that it will take about 2.9 years for WD patients from clinical symptoms to diagnosis in China. In the 242 collected surveys, the mean (*SD*) length of treatment was 12.6 ± 3.4 years. Most patients treated initially with PCA (83.5%), DMSA (62.8%), and zinc gluconate (72.7%). Half of the patients (50.8%) were treated with GDT at the same time.

**TABLE 1:** Demographic and clinical characteristics

Variable, *n* = 248	Values
Female: male ratio, *n* (%)	113 (46.7%):129 (53.3%)
Age at diagnosis, mean ± *SD* (years)	19.1 ± 7.1
Age during assessment, mean ± *SD* (years) 37.5 ± 10.4	27.9 ± 9.7
Time from symptom onset, mean ± *SD* (months)	16.2 ± 8.7
Duration of treatment, mean ± *SD* (years)	12.6 ± 3.4
Predominant clinical form at diagnosis, *n* (%)	
Hepatic	73 (30.2%)
Neurological	169 (69.8%)
Type of initial treatment, *n* (%)	
D‐penicillamine	202 (83.5%)
DMSA	152 (62.8%)
Zinc gluconate	176 (72.7%)
GDP	123 (50.8%)
Type of treatment during assessment, *n* (%)	
D‐penicillamine	189 (78.1%)
DMSA	146 (60.3%)
Zinc gluconate	170 (70.2%)
GDP	120 (49.4%)
Persistence in drug use, *n* (%)	
Yes	161 (66.53%)
No	81 (33.47) %
Adverse events, number of subjects (%) 53 (31.2)	
No	119 (49.17)
Yes, tolerable	96 (39.67)
Yes, intolerable	27 (11.16%)
Outcome of treatment (at the time of assessment), *n* (%)	
Improvement	171 (70.66%)
No change	49 (20.25)
Worsening	22 (9.09%)

Abbreviation: *SD*, standard deviation

### 3.2 Choice and combination of medicines for WD patients in China

**FIGURE 1:**
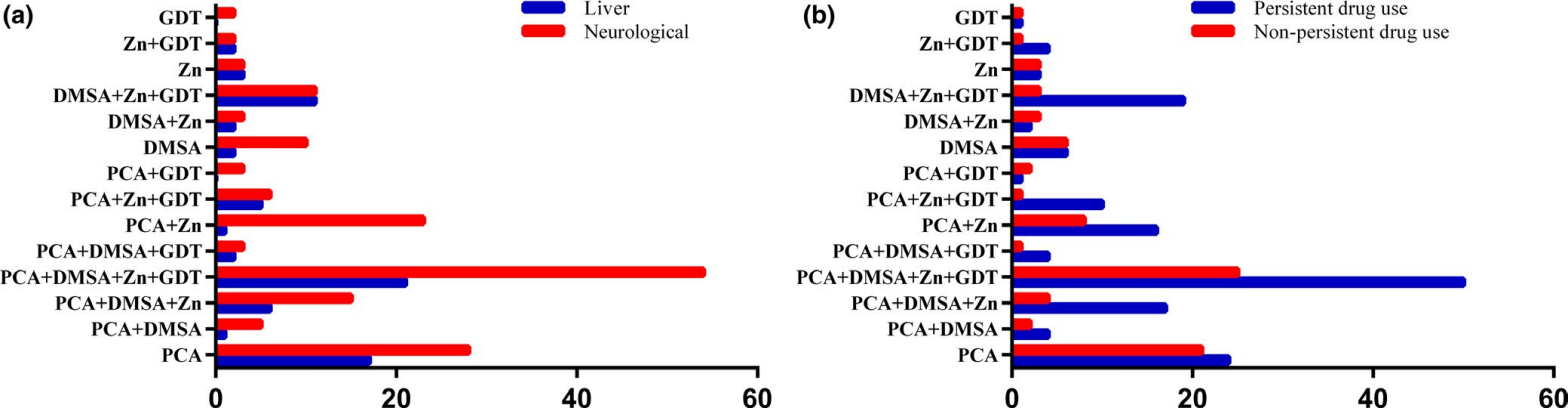
Choice and combination of medicines for WD patients in China. (a) Choice and combination of medicines in liver and neurological type WD. (b) Choice and combination of medicines in the group of PDT and n‐PDT WD. PDT, persistence drug treatment; n‐PDT, nonpersistence drug treatment

In China, besides PCA and zinc gluconate, there are also DMSA and GDT (Chinese patent medicine) which were recommended by Guideline for diagnosis and treatment of Wilson disease in China. So, the choice and the combination of medicines for WD patients are more abundant and diverse than that in western countries. In 242 collected surveys, there are 14 combined therapies for WD patients in China, PCA (54 cases), PCA+DMSA (6), PCA+DMSA+Zn (21), PCA+DMSA+Zn+GDT (75), PCA+DMSA+GDT (5), PCA+Zn (24), PCA+Zn+GDT (11), PCA+GDT (3), DMSA (12), DMSA+Zn (12), DMSA+Zn+GDT (22), Zn (6), Zn+GDT (5), GDT (2). There was no significant difference in medicine combination between the group of hepatic and group of neurological (*p* = .99). There was no significant difference in medicine combination between the group of PDT and the group of n‐ PDT (*p* = .23). Details are presented in the Figure 1.

### 3.3 Treatment outcome and AEs by treatment persistence

Of the 242 patients, 161 patients (66.5%) were divided into group of PDT and 81 patients (33.5%) were divided into group of n‐PDT. PDT was significantly better than n‐PDT on clinical outcome (*p* < .001; Table 2). In group of PDT, better outcomes were observed: improvement (78.3%) and no change‐no progression of disease (16.1%) versus those in n‐PDT (55.6%; and 28.4%, respectively). Out of 161 patients in group of PDT, only nine patients deteriorated (6.8%) in comparison with 13 patients out of 81 in group of n‐PDT (16.0%).

**TABLE 2:** Treatment outcome and adverse events by treatment persistence

	Treatment outcome, *n* (%)			Adverse events		
	Improvement (*n* = 171)	No change (*n* = 49)	Worsening (*n* = 22)	Yes, intolerable (*n* = 27)	Yes, tolerable (*n* = 96)	No (*n* = 119)
Persistent drug use (*n* = 161)	126 (78.3)	26 (16.1)	9 (5.6)	11 (6.8)	66 (41.0)	84 (52.2)
Nonpersistent drug use (*n* = 81)	45 (55.6)	23 (28.4)	13 (16.0)	16 (19.8)	30 (37.0)	35 (43.2)
*p* value for comparison of persistent versus nonpersistent drug use	<.001			<.05		

We further analyzed the AEs between the two groups. The AEs in the group of PDT were significantly less than those in the group of n‐PDT (*p* < .05; Table 2). There was no significant difference in tolerable AEs between the two groups (PDT 41.0% and n‐PDT 37.0%). Compared with the intolerable adverse event, the group of PDT was significantly lower than the group of n‐PDT (6.8%; and 19.8%, respectively).

The mean (*SD*) length of treatment was 12.6 ± 3.4 years. One hundred and twenty‐three of 242 (50.8%) reported drug‐related AEs (data not shown). Half of the 242 patients (50.8%) had adverse event, PCA accounted for 52.0% (64 cases), DMSA accounted for 21.1% (26 cases), zinc gluconate accounted for 25.2% (31 cases), and GDT accounted for 1.6% (two cases). Of the 123 patients who had AEs related to drug treatment, 96 were tolerable, and 27 were intolerable and had to stop taking related drugs. Sixty‐four cases of PCA‐related AEs were reported, including skin rash (28 cases), nausea and vomiting (16 cases), leukopenia (nine cases), thrombocytopenia (seven cases), hematuria and proteinuria (three cases), and PCA‐induced systemic lupus erythematosus (one case). Twenty‐six cases of DMSA related AEs were reported, including nausea and vomiting (14 cases), acid regurgitation (eight cases), skin ecchymosis (two case) and gingival bleeding (two case). Thirty‐one cases of zinc gluconate‐related AEs mainly included nausea and vomiting (18 cases) and abdominal pain (13 cases). Only two cases of GDT‐related AEs were diarrhea.

### 3.4 Low medication adherence in China WD patients

**FIGURE 2:**
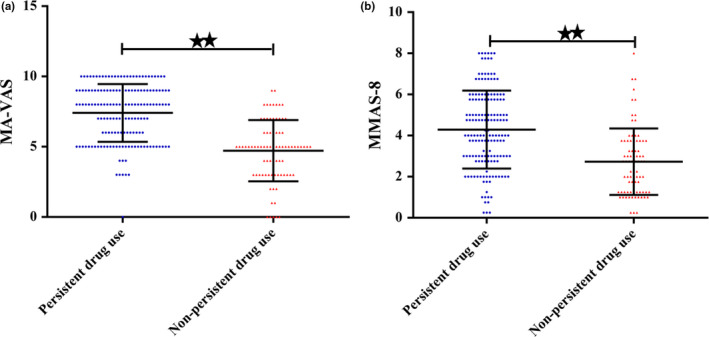
Low Medication Adherence in China WD patients. 8‐item Morisky Medication Adherence Scale (MMAS‐8) (a) and 10‐point Medication Adherence Visual Analog Scale (MA‐VAS) (b) in the group of PDT and n‐PDT WD. PDT, persistence drug treatment; n‐PDT, non‐ persistence drug treatment. ^★^ *p* < .05; ^★★^ *p* < .01

We employed the MMAS‐8 and MA‐VAS to investigate the medication adherence in WD patients. In general, WD patients in China showed lower medication adherence. Compared with the group of PDT, the group of n‐PDT showed lower medication adherence (MA‐VAS, 7.40 ± 2.05 vs. 4.74 ± 2.22, *p* < .001; MMAS‐8, 4.29 ± 1.89 vs. 73 ± 1.62, *p* < .001, respectively). Details are presented in the Table 3 and Figure 2.

**TABLE 3:** Comparison of Medication Adherence visual analogue scale and MMAS‐8 between two groups

	*n*	MA‐VAS	MMAS−8
Persistent drug use	161	7.40 ± 2.05	4.29 ± 1.89
Nonpersistent drug use	81	4.74 ± 2.22	2.73 ± 1.62
*p* value for comparison of persistent versus nonpersistent drug use		<.001	<.001

### 3.5 Relevant factors for persistence breakdown in WD

We analyzed the differences of relevant factors between the group of PDT and the group of n‐PDT, such as clinical type, age, gender, AEs, educational level, low copper diet, family knowledge about WD, and family position toward treatment (Table 4). There were no significant differences in clinical type (*p* = .66), gender (*p* = .50), age (*p* = .08), education level (*p* = .27), low copper diet (*p* = .41), and family knowledge about WD (*p* = .37) between the two groups. Compared the two groups, there were significant differences in AEs (*p* < .001) and family position toward treatment (*p* < .001; Table 4).

**TABLE 4:** Relevant factors for persistence breakdown in WD

Description	Persistent	Nonpersistent	*p* Value for persistent versus nonpersistent
Clinical type, *n* (%)			
Hepatic (*n* = 73)	47 (29.2)	26 (32.1)	.66
Neurological (*n* = 169)	114 (70.8)	55 (67.9)	
Gender, *n* (%)			
Male (*n* = 129)	83 (51.6)	46 (56.8)	.50
Female (*n* = 113)	78 (48.4)	35 (43.2)	
Age			
<10 years	13 (8.1%)	0 (0%)	.08
<20 years	35 (21.7%)	12 (14.8%)	
<30 years	54 (33.5%)	33 (40.8%)	
<40 years	48 (29.8%)	30 (37.0%)	
<50 years	8 (5.0%)	5 (6.2%)	
<60 years	3 (1.9%)	1 (1.2%)	
Adverse events, *n* (%)			
No	84 (52.2)	35 (43.2)	<.001
Yes, tolerable	66 (41.0)	30 (37.0)	
Yes, intolerable	11 (6.8)	16 (19.8)	
Educational level, *n* (%)			
Secondary school and below (*n* = 108)	66 (41.0)	42 (51.9)	.27
Upper secondary/post‐secondary (*n* = 77)	54 (33.5)	23 (28.4)	
Higher education (*n* = 57)	41 (25.5)	16 (19.7)	
Low copper diet			
Yes, strict	70 (43.5)	29 (35.8)	.41
Yes, moderate	85 (52.8)	50 (61.7)	
No	6 (3.7)	2 (2.5)	
Family knowledge about WD, *n* (%)			
Minimal (*n* = 108)	18 (11.2)	9 (11.1)	.37
Little (*n* = 108)	48 (29.8)	33 (40.8)	
Moderate (*n* = 108)	56 (34.8)	24 (29.6)	
Good (*n* = 108)	39 (24.2)	15 (18.5)	
Family position toward treatment, *n* (%)			
Supportive	133 (82.6)	53 (65.4)	<.05
Neutral	28 (17.4)	28 (34.6)	
Negative	0	0	

## 4 DISCUSSION

WD is one of the few hereditary diseases of nervous system that can be successfully treated with medicines (European Association for Study of Liver, 2012; Parkinson's Disease & Dyskinesia Group of Neurology Branch of Chinese Medical Association & Neurogenetics Group of Neurology Branch of Chinese Medical Association, 2008). But the medicines to maintain copper balance in the body need to be taken over the whole patient's life span after diagnosis (European Association for Study of Liver, 2012; Parkinson's Disease & Dyskinesia Group of Neurology Branch of Chinese Medical Association & Neurogenetics Group of Neurology Branch of Chinese Medical Association, 2008). Recent study demonstrated one quarter of WD patients could not take anti‐copper treatment regularly and this had an important negative effect on clinical outcome in Poland (Maselbas, Czlonkowska, et al., 2019). As symptoms increase, more drugs are needed. So it seriously affects the medicine adherence. WHO report shows adherence to therapies is a primary determinant of treatment success (Sabaté, 2003). So we conduct the online survey about persistence and n‐PDT on WD in China. The results demonstrated that WD patients had lower medicine adherence (persistence 66.5%; n‐ persistence 33.5%) in China, and this also had an important negative effect on clinical outcome which was the same as the results in Poland (Maselbas et al., 2019b; Maselbas, Czlonkowska, et al., 2019).

In general, WD patients in China showed lower medication adherence. One third of the patients (33.5%) were unable to PDT (Table 2), and the scores of MA‐VAS and MMAS‐8 were lower (Figure 1). But the clinical outcome was better than those in Poland (Maselbas, Czlonkowska, et al., 2019). We speculate that it may be related to the more medicines taken by WD patients in China. Besides PCA and zinc gluconate, there also had DMSA and GDT (Chinese patent medicine) which were recommended by Guideline for diagnosis and treatment of Wilson disease in China (Parkinson's Disease & Dyskinesia Group of Neurology Branch of Chinese Medical Association & Neurogenetics Group of Neurology Branch of Chinese Medical Association, 2008).

There was no significant difference in the choice and the combination of medicines among patients with different clinical types, and there was also no significant difference between the group of PDT and the group of n‐PDT. So we need to further develop drugs that take fewer times (such as sustained release agents), more convenient (transdermal absorption patches) to treat WD.

Of the 242 patients, 171(70.7%) improved which was higher than this (22.4%) in Poland (Maselbas, Czlonkowska, et al., 2019). In addition, our results were significantly lower in patients with clinical exacerbations than this in Poland (9.1%; and 15.3%, respectively) (Maselbas, Czlonkowska, et al., 2019). In Poland, PCA is the main medicine for WD patients. Recent studies have demonstrated that some patients can be worsened following metal chelating agents, especially PCA (Ranjan et al., 2015; Kalita et al., 2014, 2015). DMSA, a broad‐spectrum metal antidote which was recommended for WD (Parkinson's Disease & Dyskinesia Group of Neurology Branch of Chinese Medical Association & Neurogenetics Group of Neurology Branch of Chinese Medical Association, 2008), was first used to treat WD in China (Yang & Zhang, 1990). In China, DMSA is recommended for patients with neurological and psychiatric symptoms, mild–moderate liver symptoms, and for those who are not tolerant of PCA (Parkinson's Disease & Dyskinesia Group of Neurology Branch of Chinese Medical Association & Neurogenetics Group of Neurology Branch of Chinese Medical Association, 2008). DMSA for WD is Chinese characteristic and the medicine was not recommended in the practice guidelines published by the American Association for the Study of the Liver Diseases (Roberts & Schilsky, 2008) and the European Association for the Study of the Liver (European Association for Study of Liver, 2012). Compared with PCA, DMSA had less AEs and less deterioration (Parkinson's Disease & Dyskinesia Group of Neurology Branch of Chinese Medical Association & Neurogenetics Group of Neurology Branch of Chinese Medical Association, 2008). In addition, PCA is not recommended for WD patients with obvious neurological symptoms in China, such as severe limb torsion and dysphagia (Parkinson's Disease & Dyskinesia Group of Neurology Branch of Chinese Medical Association & Neurogenetics Group of Neurology Branch of Chinese Medical Association, 2008). GDT, as a representative Traditional Chinese Medicine for WD, promotes the excretion of copper in the bile, feces, and urine (Li et al., 2013). A systematic review indicated that traditional Chinese medicine as a monotherapy or an adjuvant therapy generally appears to be effective, safe, and well‐tolerated (Wang et al., 2012). Therefore, we speculate that although PCA is still the main medicine for WD patients in China, the alternative oral medicine of DMSA and GDT may reduce the symptom deterioration in some patients.

We further analyzed the factors that may be related to treatment persistence, we did not find any impact of clinical type, gender, age, education level, low copper diet, and family knowledge about WD. In theory, we would like to think that people with high education level will have better medicine adherence. However, although 41 (25.5%) WD patients received higher education, 54 (33.5%) received upper secondary/post–secondary education, and the statistical results show that there is no statistical difference between education level and PDT for WD in China. Our results indicated that education level had no related to treatment persistence, which is different from the results of the Polish study (Maselbas et al., 2019b; Maselbas, Czlonkowska, et al., 2019). We also think that older patients and family with more knowledge about WD will have better drug compliance, but there also no statistical difference between the two groups. But we find AEs (*p* < .001) and family position toward treatment (*p* < .05) had impact on treatment persistence. WD patients with anti‐copper treatment should be monitored according to copper metabolism and general health parameters (especially hematologic and liver function tests with periodic neuropsychiatric assessment) to ensure that patients are treated correctly, verify patient compliance with WD treatment, and avoid drug‐related adverse reactions or overtreatment (Członkowska, 2017. Therefore, we need to further develop metal chelating agents with low or no AEs to treat WD.

Our study has some limitations. Firstly, it is a retrospective online survey study. Although WeChat is the most popular social software in China, some elderly patients or patients without smart phones will not be investigated, and so the data are missing to some extent. Secondly, we did not investigate dead patients. So the data of clinical outcome are defective. Thirdly, due to the online self‐report survey, the design of related issues is simpler and cannot further reflect the real situation of patients. Finally, we failed to assess the clinical situation of patients in combination with copper metabolism.

## 5 CONCLUSIONS

In summary, our results indicate that patients with WD have low medicine adherence and treatment persistence is a pivotal factor for a positive clinical outcome for WD in China. The choice and the combination of medicines for Chinese WD patients are more abundant and diverse and have higher clinical improvement rate and lower clinical deterioration rate. AEs and a positive, supportive family position toward WD treatment seem to be the most important factors that may impact treatment persistence for WD patients in China.

## CONFLICT OF INTEREST

None declared.

## AUTHOR CONTRIBUTIONS

ZHZ carried out statistical analysis and drafted the manuscript. ZZH, MFH, and GQW participated in the study design, data collection, and interpretation of the data. YFW, YY, AQL, YQH, QYY, and ZXP carried out data collection, statistical analysis, and interpretation of data. All authors read and approved the final manuscripts.

## ETHICAL APPROVAL AND CONSENT TO PARTICIPATE

The principles of the Declaration of Helsinki were followed, and this study was approved by the ethics committee of the first affiliated hospital, school of clinical Medicine of Guangdong Pharmaceutical University.

## CONSENT FOR PUBLICATION

All the authors have approved the manuscript.

### PEER REVIEW

The peer review history for this article is available at https://publons.com/publon/10.1002/brb3.2168.

## Data Availability

All the data mentioned in this article are available on published article.
